# Authentication of Primordial Characteristics of the CLBL-1 Cell Line Prove the Integrity of a Canine B-Cell Lymphoma in a Murine *In Vivo* Model

**DOI:** 10.1371/journal.pone.0040078

**Published:** 2012-06-28

**Authors:** Barbara C. Rütgen, Saskia Willenbrock, Nicola Reimann-Berg, Ingrid Walter, Andrea Fuchs-Baumgartinger, Siegfried Wagner, Boris Kovacic, Sabine E. Essler, Ilse Schwendenwein, Ingo Nolte, Armin Saalmüller, Hugo Murua Escobar

**Affiliations:** 1 Central Laboratory, Department of Pathobiology, University of Veterinary Medicine Vienna, Vienna, Austria; 2 Small Animal Clinic and Research Cluster of Excellence ‘REBIRTH’, University of Veterinary Medicine Hannover, Hannover, Lower Saxony, Germany; 3 VetBioBank, VetCore Facility for Research, University of Veterinary Medicine Vienna, Vienna, Austria; 4 Institute of Pathology, Department of Pathobiology, University of Veterinary Medicine Vienna, Vienna, Austria; 5 Department of Biomedical Sciences, Translational Oncology, Institute for Animal Breeding and Genetics, University of Veterinary Medicine Vienna, Vienna, Austria; 6 Department of Pathobiology, Institute of Immunology, University of Veterinary Medicine Vienna, Vienna, Austria; National Cancer Institute, United States of America

## Abstract

Cell lines are key tools in cancer research allowing the generation of neoplasias in animal models resembling the initial tumours able to mimic the original neoplasias closely *in vivo*. Canine lymphoma is the major hematopoietic malignancy in dogs and considered as a valuable spontaneous large animal model for human Non-Hodgkin's Lymphoma (NHL). Herein we describe the establishment and characterisation of an *in vivo* model using the canine B-cell lymphoma cell line CLBL-1 analysing the stability of the induced tumours and the ability to resemble the original material. CLBL-1 was injected into Rag2^−/−^γ_c_^−/−^ mice. The generated tumor material was analysed by immunophenotyping and histopathology and used to establish the cell line CLBL-1M. Both cell lines were karyotyped for detection of chromosomal aberrations. Additionally, CLBL-1 was stimulated with IL-2 and DSP30 as described for primary canine B-cell lymphomas and NHL to examine the stimulatory effect on cell proliferation. CLBL-1 *in vivo* application resulted in lymphoma-like disease and tumor formation. Immunophenotypic analysis of tumorous material showed expression of CD45^+^, MHCII^+^, CD11a^+^ and CD79αcy^+^. PARR analysis showed positivity for IgH indicating a monoclonal character. These cytogenetic, molecular, immunophenotypical and histological characterisations of the *in vivo* model reveal that the induced tumours and thereof generated cell line resemble closely the original material. After DSP30 and IL-2 stimulation, CLBL-1 showed to respond in the same way as primary material. The herein described CLBL-1 *in vivo* model provides a highly stable tool for B-cell lymphoma research in veterinary and human medicine allowing various further *in vivo* studies.

## Introduction

Development of *in vitro* and *in vivo* models able to recapitulate the natural history of cancers and their clinical response to therapy is an important prerequisite for rapid bench-to-bedside translation of anticancer therapies [1]. While early stage evaluations can be done *in vitro* using cell lines, the more complex experimental tasks require the establishment of tumor specific *in vivo* animal models. Both systems show specific advantages and disadvantages limiting the respective experimental possibilities. Cell lines are widely used to generate animal *in vivo* tumor models in rodents leading to significant results.

Despite of all the striking achievements generated in these *in vivo* rodent models, some major tumor characteristics of naturally occurring tumors cannot be provided by experimentally induced tumors or tumors transplanted into immunocompromised animals. Thus, spontaneous occurring tumor models have been lately attracting significant interest in cancer research completing the well-established conventional *in vitro* and *in vivo* models [2].

In this context, canine tumors have been considered as valuable naturally occurring models helping to reveal mechanisms in cancer development and behaviour. The neoplasias seen in dogs arise spontaneously and have been described to mimic human tumors in many ways as e.g. tumor progression, metastatic pattern, and histology [3]. These facts suggest that also the mechanisms of tumor development could be similar in large parts between man and dog, as for example the development of the canine neoplasias occurs with the background of an intact immune defence and tumor evasion. Consequently, the combination of *in vitro* and *in vivo* models with spontaneously occurring primary samples and veterinary patients like the dog bear major advantages for cancer research. However, before engaging veterinary patients, *in vitro* and *in vivo* rodent models able to reproduce the tumors as they occur in dogs will be needed and thus be of great value. Despite of these advantages the major critical point in this is the capacity of the induced tumors to mimic the original neoplasia/cell line as close as possible *in vivo* and thus these characteristics have to be evaluated critically.

Focusing on hematopoietic tumors, canine lymphoma is considered a useful translational model to study the pathogenesis and treatment of lymphoma due to the fact that dogs share extensive genome homology with humans [4], [5], [6], [7], [8], [9], [10], [11], [12], [13], [14], [15]. The frequency of canine lymphoma cases in hematopoietic malignancies is approximately 83% representing 7% to 24% of all canine neoplasms [16]. The response of this malignancy to chemotherapy protocols varies substantially [17] and in contrast to humans, the remission time in canine lymphoma is much shorter. This compressed clinical course of disease reduces the time required to perform longitudinal studies.

In the present study, we show that Rag2^−/−^γ_c_^−/−^ mice injected subcutaneously with the canine lymphoma cell line CLBL-1 [18] develop multicentric lymphoma as observed in canine patients. Affected organs of the inoculated mice show stable expression of intra- and extracellular markers in immunophenotyping, antigen receptor rearrangement, and histology. We derived the cell line CLBL-1M from the generated tumor material and comparatively characterised the karyotype of CLBL-1 and CLBL-1M revealing a strong chromosomal stability. Further, we could show that CLBL-1 responds in the same way as primary material to DSP30 and IL-2 stimulation. This represents a stable model of canine diffuse large B-cell lymphoma (DLBCL) that closely resembles the disease as it occurs in dogs and humans giving the opportunity for more accurate preclinical evaluation of investigational therapies against lymphoma.

## Materials and Methods

### Cultivation of the CLBL-1 B-cell Lymphoma Cell Line

The cell culture conditions, as well as the characteristics of the canine B-cell lymphoma cell line CLBL-1 have been described previously [18].

### Animals and Lymphoma Cell Inoculation

#### Animals

Immunodeficient Rag2^−/−^γ_c_^−/−^ mice [19], [20] were maintained under specific pathogen-free conditions in the animal facility of the Institute of Animal Breeding and Genetics, Department of Biomedical Sciences, University of Veterinary Medicine Vienna, Vienna, Austria. All animal experiments were performed according to the rules of the Austrian Animal Law 1989/2005, licence number 66.009/0065l-il 0b/2009 to Dr. Veronika Sexl. The mice were kept under sterile conditions in individually ventilated IVC-cages.

After subcutaneous (*s.c.*) inoculation of the cell line in the right and left flank of the animals (n = 3), these were observed on a daily basis and sacrificed by cervical dislocation depending on their clinical status. Beside the clinical status, the size of the tumor was an additional criterion for euthanasia by allowing the tumors to grow up to a size of 0.9 cm.

#### Inoculation of cells

The CLBL-1 cells were harvested from the cell culture vessel and after a washing step with RPMI 1640 medium (PAA, Pasching, Austria) supplemented with 10% heat inactivated fetal calf serum (FCS) (PAA) and penicillin 100 U/ml/streptomycin 0.1 mg/ml (PAA). After counting in an ADVIA120 haematology Analyser (Siemens, Austria) the cell pellet was washed twice in PBS without Ca2^+^ and Mg2^+^ (PAA, Pasching, Austria). The cells were resuspended in PBS with adjusting the cell count to 1×10^6^ à 100 µl. Immediately before application the cell suspension was carefully resuspended and aspirated in 1 ml syringes (Omnifix-F1ml, B. Braun, Germany) and 1×10^6^ cells were inoculated *s.c.* into the right and left flank of the animals using a 27G3/4′′ needle (Sterican, B. Braun, Germany). All mice were female (4–6 weeks old).

### Necropsy, Morphology and Histological Staining

After sacrificing the animals, necropsy was performed immediately on all individuals. Liver, spleen, bone (hind legs), solid tumorous material from the flank and macroscopically changed material of ovaries and uterus of affected animals were removed. One femur and pieces of liver and spleen, tumour, uterus and ovaries were fixed in 4% neutral buffered formaldehyde. The femur was additionally decalcified and all samples were paraffin embedded, cut into 3 µm sections and stained with haematoxylin and eosin. Immunohistochemical staining was performed on paraffin sections with a commercial antibody (Dako, Glostrup, Denmark) for identification of B cell lineage (mouse anti human CD79αcy, dilution 1∶4000).

### Generation of CLBL-1 Mouse Tumor Derived Cell Line CLBL-1M

The tumor tissue sample from the flank was cut into small pieces using a sterile scalpel. Following, the dissected tissue was transferred into a sterile 25 cm^2^ cell culture flask and treated with 3 ml collagenase (0.26%, Collagenase NB8, Serva GmbH, Heidelberg, Germany) for 2 hours at 37°C. After incubation, the dissociated cells were transferred into a sterile 10 ml tube and centrifuged for 10 min at 1000 rpm. After centrifugation the supernatant was discarded. The resuspended cell pellet was transferred into a sterile 25 cm^2^ cell culture flask and incubated in 5 ml RPMI 1640 culture medium (Biochrom, Berlin, Germany) with 20% FCS (fetal calf serum; PAA, Pasching, Austria) 200 U/ml penicillin and 200 ng/ml streptomycin (Biochrom, Berlin, Germany) in 5% CO_2_ at 37°C.

The cells were controlled in a daily routine. Free floating cell clusters of well-grown culture flasks were subcultivated in upright standing 75 cm^2^ cell culture flasks with 20 ml RPMI twice a week. The growth curve and the population doubling time were evaluated and calculated using the method reported previously in Rütgen et al. 2010 [18].

### Cell Surface and Intracellular Marker Analysis

For analysis of the cells of the cell line CLBL-1 inoculated in the animals cells derived from the tumorous material were labelled with anti-canine or anti-human cross-reactive monoclonal antibodies (mAb) listed in Table 1. Most of these mAb were directly conjugated with fluorochromes (see Table 1 for details). For each analysis 1×10^6^ cells were labelled as described previously [18].

**Table 1:** Monoclonal antibodies used for flow cytometry for CLBL-1 before inoculation of the cells, after sacrificing the inoculated mice and after cultivating the isolated cells *in vitro* (CLBL-1M).

	Clone	Isotype	Fluorescence labelling
CD3	CA17.2A12	mIgG1	FITC
CD5	YKIX322.3	rIgG2a	FITC, PE
CD8	YCATE 55.9	rIgG1	PE
CD11a	HI111	mIgG1	APC
CD21	B-ly-4	mIgG1	APC
CD21 like (anti B-Cell)	CA2.106	mIgG1	anti-mouse IgG1-PE*
CD25	P4A10	mIgG1	PE
CD45	YKIX716.13	rIgG2b	APC
CD45RA	CA4.1D3	mIgG1	anti-mouse IgG1-PE*
CD56	MOC-1**	mIgG1	PE
CD79αcy	HM57	mIgG1	PE
MHC II	YKIX334.2	rIgG2a	FITC

* Fluorescence labelling was achieved by use of a secondary antibody.

Abbreviations: m  =  mouse; r  =  rat; FITC  =  Fluorescein isothiocyanate, APC  =  Allophycocyanin;

PE  =  Phycoerythrin.

** cross-reactivity pattern for canine lymphocytes lymphocytes [36].

The labelled cells were analysed on a FACSCanto II flow cytometer (BD Biosciences, San Jose, CA, USA) immediately after staining. For intracellular staining, the IntraStain-Kit (Dako, Glostrup, Denmark) was used according to manufacturers’ instructions.

For analysis of the cells, tumor material obtained from the mass in the flank was tested for cell surface and intracellular markers expressed by the inoculated cell line by flow cytometry (FCM). Using a reduced cell number of 5×10^5^ cells per tube, the staining protocol was the same like for the cell line mentioned above. One additional step was used to block unspecific binding of the antibodies using Chrome pure mouse IgG (Jackson ImmunoResearch Laboratories, West Grove PA, USA) before labelling with the first antibodies. Erythrocytes were lysed using ADG lyse (An der Grub, Austria) after incubation with the first mAb.

FCM staining of the tumor was performed using a panel of antibodies, already used in the antibody panel for the cell line and verifying the canine origin of the cells as the lymphoma cell line CLBL-1: mouse anti-human CD3:FITC (Clone: CA17.2A12, Serotec), rat anti-canine CD5:PE (Clone: YKIX322.3, Serotec), mouse anti-canine CD11a:APC (Clone: HI111, BD), rat anti-canine CD45:APC (Clone: YKIX716.13, Serotec), mouse anti-human CD79αcy:PE (Clone: HM57, dako) and rat anti-canine MHCII:FITC (Clone: YKIX334.2, Serotec), (see Table 1).

The cells of the CLBL-1 and the CLBL-1M cell line were labelled the same way as described above using the anti-canine or anti-human cross-reactive mAb (Table 1).

### Polymerase Chain Reaction for Antigen Receptor Rearrangements (PARR)

For the polymerase chain reaction for antigen receptor rearrangement (PARR) assay, total genomic DNA was extracted from CLBL-1 cells, cells obtained from the induced tumorous mass located in the flank of one inoculated Rag2^−/−^γ_c_^−/−^ mouse and from the mouse tumor derived cell line CLBL-1M using a commercial kit following the manufacturer’s instructions (GenEluteTM Mammalian Genomic DNA Miniprep Kit, Sigma, Vienna, Austria) including negative extraction controls. The DNA was eluted with 200 µl elution buffer supplied with the kit. A 5 µl aliquot was analyzed on a 0.7% DNA grade agarose gel (Fisher Scientific, Schwerte, Germany) and visualised after staining with GelRedTM (Biotium, Hayward, CA, USA) together with a High Range DNA Ladder (GeneRulerTM High Range DNA Ladder, ready-to-use; Fermentas, Burlington, Ontario, Canada). The DNA samples were assayed by amplifying the Cµ DNA control [21], the immunoglobulin heavy chain (IgH) gene rearrangements with the primer sets CB1, CB2 and CB3 [21] and the T-cell receptor gamma (TCRγ) gene rearrangements with the primer sets DPA, DPB and DPC [22]. The PCR mixture was composed of 1× Type-it® Multiplex PCR Master Mix (Qiagen GmbH, Hilden, Germany), 1× Q-Solution® (Qiagen), 1× CoralLoad® (Qiagen), 0.2 mM of each nucleotide (PCR Grade; Promega, Madison, Wis.), 10 pM of each primer (Eurofins MWG Operon, Ebersberg, Germany) and 100 ng of eluted genomic DNA as template brought up to 50 µl with molecular biology grade water (Sigma). Each PCR reaction was carried out in duplicate including positive and negative PCR controls in each PCR run. The PCR reactions were carried out using a T Gradient thermal cycler (Biometra, Göttingen, Germany) and the thermal cycling conditions were 94°C for 3 min, followed by 35 cycles at 94°C for 30 s, 60°C for 20 s and 72°C for 20 s. After PCR, the amplicons were first analysed on a 4% low melting Phor agarose gel (Biozym Biotech Trading GmbH, Vienna, Austria) and visualised after staining with GelRedTM (Biotium) together with a 100-bp Ladder (O’GeneRulerTM 100 bp DNA Ladder, ready-to-use, Fermentas). To verify the clonality of IgH gene rearrangements, the obtained PCR products were cloned using the pGEM-T Easy vector system (Promega) and 16 positive bacterial clones of each transformation were subjected to automated sequencing with M13 standard sequencing primer (Eurofins MWG Operon).

### Cytogenetic Analysis CLBL-1 and CLBL-1M Cells

For chromosome preparation the CLBL-1 and CLBL-1M cells were cultured in RPMI 1640 as described above. Half the cell material of a well-grown 75 cm^2^ culture flask was used for one chromosome preparation. Colcemid was added at a final concentration of 0.1 µg/ml for 2 h before harvesting. Subsequently, the cells were incubated for 20 min in hypotonic medium (1 : 7; RPMI : H_2_O) in 15 ml conical bottom centrifugation tubes (Greiner Bio-One, Frickenhausen, Germany) on a MACSmix Tube Rotator (Miltenyi Biotec GmbH, Bergisch Gladbach, Germany) with 12 rpm rotation and finally fixed with methanol/glacial acetic acid (3∶1) following routine methods [23]. The suspension was dropped on ice-cold slides and dried for 5 days at 37°C followed by GTG-banding which was performed as previously described by [15]. Results were processed and recorded with BandView, 6.0, MultiSpecies, Applied Spectral Imaging, Israel. Karyotype description followed the nomenclature proposed by Reimann et al. [24].

### Quantitative Real-time PCR Analyses for Canine *MYC* Expression in CLBL-1 and CLBL-1M

Following samples were used for quantitative real-time PCR analyses: the herein in detail analysed cell lines CLBL-1 and CLBL-1M, DT08/40 derived from canine neoplastic tissue characterised by polysomy of CFA13, and a non-neoplastic FNA lymph node reference sample. All samples were homogenised with QIAshredder columns accordingly to the manufacturer’s protocol (Qiagen, Hilden, Germany).

#### RNA isolation and cDNA syntheses

RNAs from the lymph node sample and cultured cell lines were isolated using the RNeasy Mini Kit according to the manufacturer’s instructions (Qiagen, Hilden, Germany). To avoid genomic DNA contaminations, on-column DNase digestion with the RNase-Free DNase set (Qiagen, Hilden, Germany) was performed. cDNA syntheses were done using 250 ng RNA and the QuantiTect Reverse Transcription Kit following the manufacturer’s protocol (Qiagen, Hilden, Germany).

#### MYC and HPRT real-time PCR

Relative quantification of the canine *MYC* and *HPRT* genes were carried out using the Eppendorf Mastercycler ep realplex real-time PCR System (Eppendorf AG, Hamburg, Germany).

For analysis of the human target genes, 2 µl of each cDNA were amplified in a total volume of 20 µl using universal PCR Mastermix and TaqMan gene Expression Assays for canine *MYC* (Assay ID: cf02628821_m1) and *HPRT (Assay ID:* cf02626254_g1) (Applied Biosystems, Darmstadt, Germany). PCR conditions were as follows: 2 min at 50°C and 10 min at 95°C, followed by 40 cycles with 15 s at 95°C and 1 min at 60°C.

All samples were measured in triplicates further non-template controls and non-reverse transcriptase control reactions were included.

A precedent efficiency analysis of all PCR assays used in this study was performed by applying the same templates and dilutions. Statistical analysis of relative expression of real-time PCR results was done by using the software tool REST 2009, version 2.0.13. A p-value of ≤0.05 was considered as statistically significant.

### DSP30/IL-2 Stimulation

CLBL-1 and CLBL-1M cells (5×10^4^ cells/well in 100 µl RPMI 1640) were stimulated in 96-well flat bottom plates with 0.5, 1.0 and 5.0 µmol/l CpG-oligonucleotide DSP30 (TIBMolBiol, Berlin, Germany) and 50, 100 and 500 U/ml human interleukin-2 (IL-2) (Peprotech GmbH, Hamburg, Germany) in single applications and in combination (0.5 µmol/l DSP30 combined with 50 U/ml IL-2; 1.0 µmol/l DSP30 combined with 100 U/ml IL-2; 5.0 µmol/l DSP30 combined with 500 U/ml IL-2).

The stimulation was performed for 24, 48, 72 and 96 h for each respective stimulation experiment. Each experiment was performed in eight replicates.

### Cell Proliferation Assay

Proliferation of cells in response to DSP30 and IL-2 stimulation was evaluated using a colorimetric cell proliferation ELISA (Roche Applied Science, Mannheim, Germany). This assay measures the incorporation of the thymidine analogue 5-bromo-2-deoxyuridine (BrdU) into newly synthesised DNA of replicating cells by ELISA using an anti-BrdU monoclonal antibody.

The proliferation assay was carried out according to the manufacturer’s protocol for suspension cells (Cell proliferation ELISA, colorimetric, Roche Applied Science, Mannheim, Germany). The reaction products were quantified by measuring the absorbance at 370 nm (reference wavelength 492 nm) with a maximum of 27 single reads over a time period of 30 min using a scanning multiwell spectrophotometer equipped with the analysis software Gen 5 (Synergy HT multi-mode microplate reader, BioTek Instruments Inc., Bad Friedrichshall Germany). The absorbance results directly correlate to the amount of DNA synthesis and hereby to the number of proliferating cells.

Results are stated as mean absorbance values expressed as Max V [delta 370–492]. The proliferation results of each stimulation experiment were compared to the corresponding unstimulated reference cells.

### Statistics

Results are presented as mean ± standard deviation. The Shapiro-Wilk test was applied to test if the data are normally distributed using OriginPro 8 software (OriginLab Corporation, Northampton, USA). Based on the outcome of the Shapiro-Wilk test, the non-parametric one-tailed Wilcoxon matched pairs signed rank test for related samples was performed to assess the significance of differences between stimulated and unstimulated cells using GraphPad Prism Software (GraphPad Software, La Jolla, USA). Differences were considered statistically significant for *p≤0.05, **p≤0.001 to 0.01 and ***p<0.001.

## Results

### Application of CLBL-1 Cells, Tumorigenicity and Growth in Rag2^−/−^γ_c_^−/−^ Mice

CLBL-1 cells were highly tumorigenic in Rag2^−/−^γ_c_^−/−^ mice. In all of the subcutaneously inoculated mice (n = 3) the acute lymphoma-like disease strongly affected liver, spleen, bone marrow, ovaries and uterus. All mice were sacrificed on day 25 showing tumorous masses at the sites of injection (1/3), abdominal distention (2/3) and depressed behaviour with reduced level of activity and tarnished looking fur (3/3).

In a preliminary study 2 mice were used as sham control mice showing no clinical or pathological signs [25].

### Post Mortem Examination of the CLBL-1 Inoculated Mice

**Figure 1:**
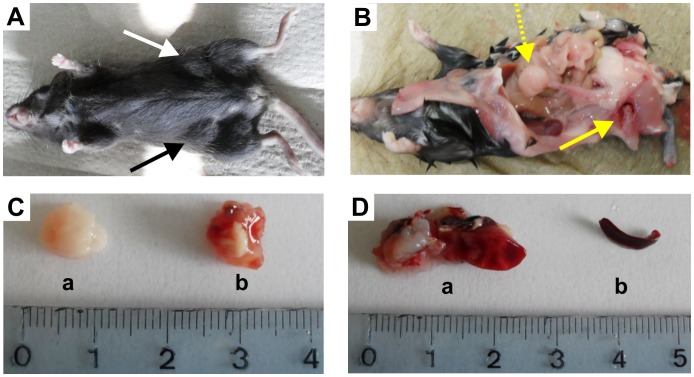
Images of immunodeficient Rag2^−/−^γ_c_^−/−^ mice after *s.c.* inoculation of the cell line CLBL-1: (A) image of on representative mouse with solid tumours located at the site of injection (see two arrows black and white) (picture taken from inoculated mouse CLBL1-I), (B) situs of on representative mouse with solid tumours located at the site of injection (see solid lined arrow) and enlarged ovary and uterus (dotted lined arrow) (picture taken from inoculated mouse CLBL-1 I), (Ca) ovary (picture taken from inoculated mouse CLBL-1 I) (Cb) tumour mass from injection site (picture taken from inoculated mouse CLBL-1 I) (D) spleen of two inoculated mice with different extent of infiltration (picture taken from inoculated mouse CLBL-1 II (Da) + CLBL-1 III (Db).

Macroscopically, livers were enlarged, but did not show tumor masses in 3/3 individuals. Spleen was enlarged in 2/3 individuals (Fig. 1D), and the uterus and ovaries were enlarged in 2/3 affected mice (Fig 1Ca). Two out of three mice showed abdominal distension pointing to the enlarged spleen, ovaries and uterus and one mouse (1/3) showed bilateral distensions on the right and the left flank indicating solid tumor masses referring to the site of injection (Fig. 1A, 1Cb). All the other abdominal and thoracic organs (gastrointestinal tract, kidneys, urinary tract, lung) examined did not show any evidence of the infiltrating lymphoid population.

**Figure 2:**
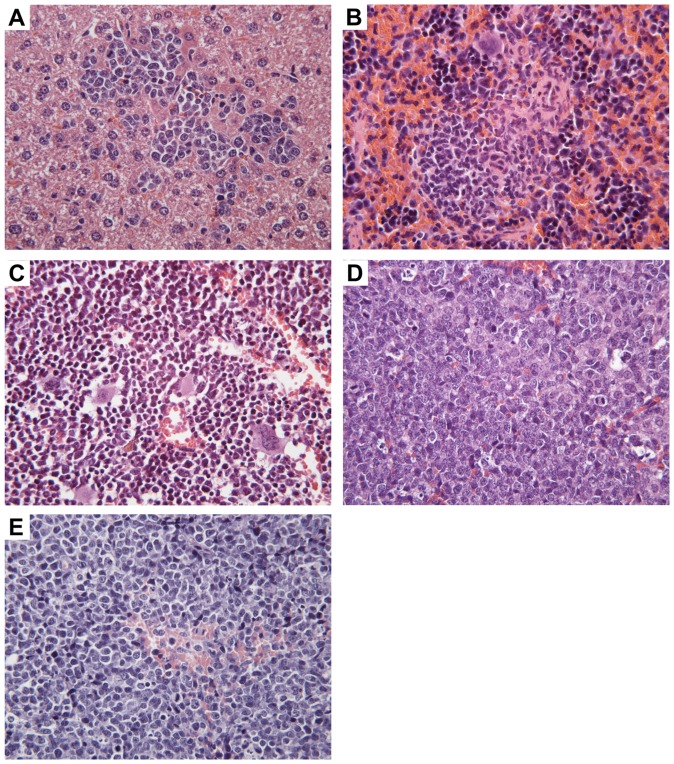
Morphologic characteristics of the CLBL-1 cell line as a xenograft tumour in Rag2^−/−^γ_c_^−/−^ mice. Neoplastic infiltrative cells in the liver (A), spleen (B), bone marrow (C), ovary (D) and solid tumour mass at injection site (E); (H&E, magnification×400, pictures taken from inoculated mouse CLBL-1 I).

Pathohistological examination revealed disseminated infiltrates of lymphoid cells in liver (3/3), spleen (2/3), bone marrow (1/3), uterus and ovaries (2/3) (Fig. 2A, B, C, D) and solid tumours bilaterally of the s.c. sites of injection (0.9 cm Ø) in one out of three mice (Fig. 2E). The lymphoid cells had round nuclei with 2–2.5 red cells in diameter. The chromatin was granular or branched. The nuclei showed a single central nucleolus or multiple nucleoli nearby the nuclear membrane. The mitotic rate was high with 6–9 mitoses/400x field. In the subcutaneous tumorous mass and in one spleen necrosis of small cell groups was evident whereas in the other localizations single cell necrosis could be observed. These pathohistologic characteristics refer to a high grade lymphoma.

**Figure 3:**
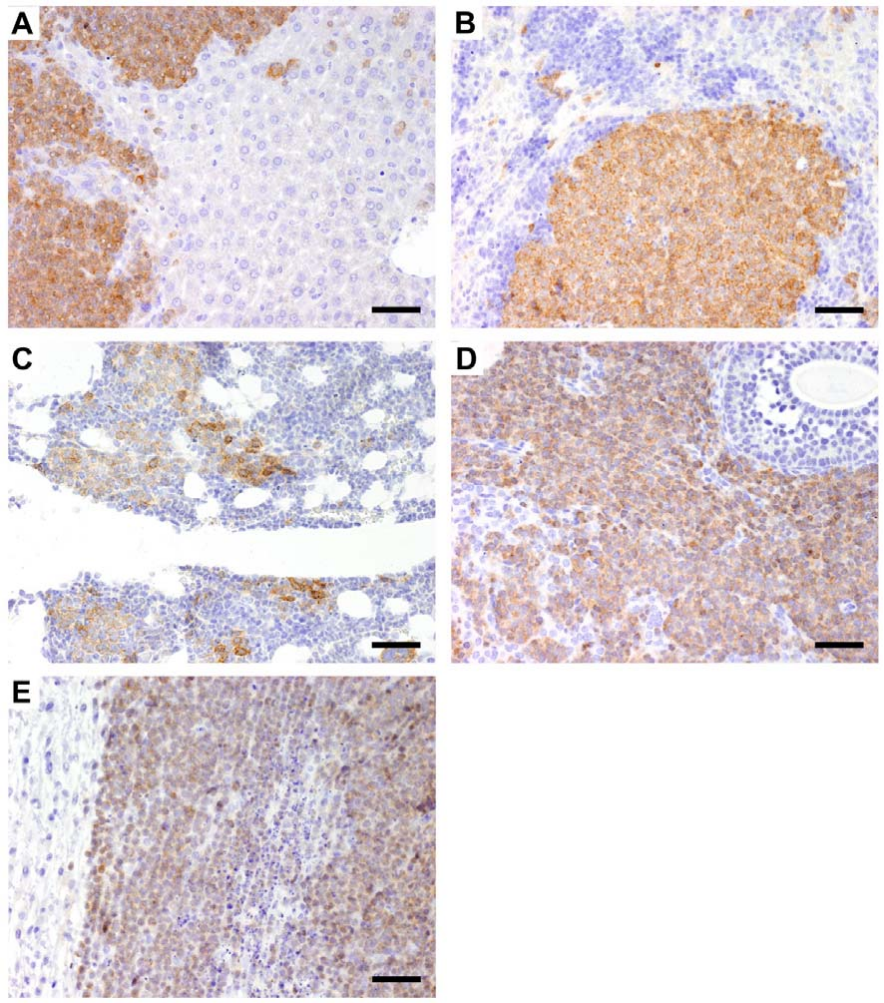
CD79αcy expression of the CLBL-1 cell line as a xenograft tumour in Rag2^−/−^γ_c_^−/−^ mice. Neoplastic B cells referring to the characteristics of the inoculated cell line infiltrating the liver (A), spleen (B), bone marrow (C), ovary (D) and solid tumour mass at injection site (E); (H&E, magnification ×200, pictures A, D, E taken from inoculated mouse CLBL-1 I; pictures B,C taken from inoculated mouse CLBL-1 II), bar  =  80 µm.

Positive staining for CD79αcy in IHC confirmed the phenotype of the CLBL-1 cell line used for injection in liver (3/3), spleen (2/3), bone marrow (1/3), uterus and ovaries (2/3) and tumor material (1/3) (Fig. 3A, B, C, D, E).

### Immunophenotyping of Tumorous Tissue of Inoculated Rag2^−/−^γ_c_^−/−^ Mice

**Figure 4:**
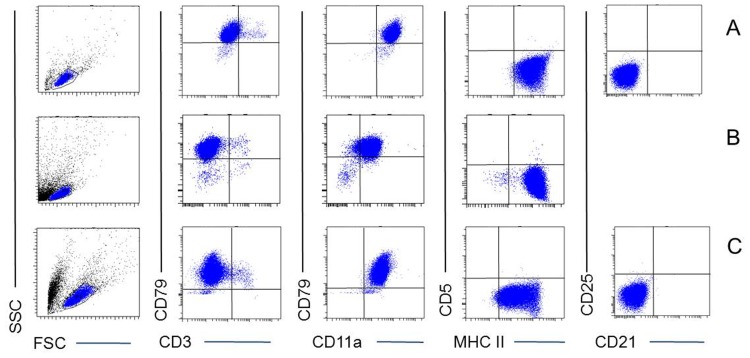
FCM analyses of the CLBL-1 cell line (A) and tumorous material derived from the site of inoculation of CLBL-1 cells (B). In the left row dot-plots depicting forward/side scatter (SSC/FSC) signals are shown to illustrate gating of the respective lymphocyte populations. Dot-plots in the three right rows show expression of various antigens of the gated lymphocyte population. Vertical and horizontal lines in the dot plots mark the boundaries between positive and negative cells for the respective markers as established by corresponding isotype control samples. Per sample at least 1×10^4^ of gated cells were analysed.

FCM analysis confirmed the presence of MHCII^+^ (98.9%) and CD11a^+^CD79αcy^+^ (95.7%) cells (Figure 4B) in the tumorous material of the right flank. All cell populations were negative for CD5 and CD3 confirming the phenotype of the parental CLBL-1 cell line initially used for injection (Figure 4B). The CLBL-1 cells analysed on the day of inoculation of the cells showed expression of MHCII^+^ (99.8%) and CD11a^+^CD79αcy^+^ (99.3%) (Fig. 4A) were additionally positive for CD45, CD45RA and negative for CD8, CD21, CD21 like and CD56 (data not shown) confirming their stable phenotype as published in Rütgen et al., 2010 [18]. The CLBL-1 cells were additionally negative for CD25 (Fig. 4A).

### Clonal IgH Gene Rearrangement of the CLBL-1 Cells and the CLBL-1 Induced Tumor - PARR Analysis

**Figure 5:**
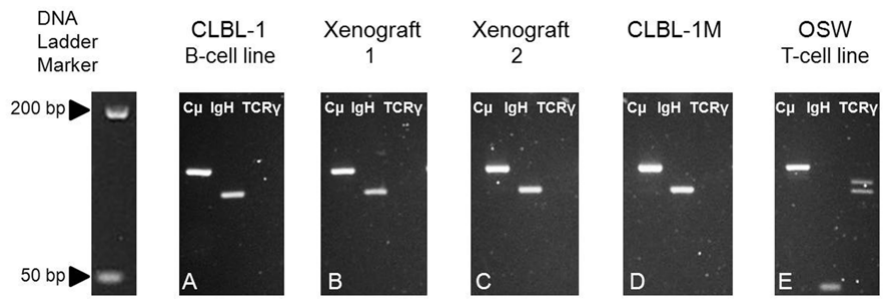
PCR for antigen receptor rearrangement. PARR of CLBL-1 cells (A), Xenograft-1 (tumor of right flank,B), Xenograft-2 (tumor of right flank,C), CLBL-1M cells (D) and the OSW cell line (E). Lane 1 shows Cµ serving as positive control for DNA. Lanes 2 and 3 show bands of IgH and TCRγ PCR products, respectively.

CLBL-1 and two representative cell samples from one solid tumor of the right flank used for the establishment of the cell line CLBL-1M were analysed by PCR for TCRγ and IgH gene rearrangements yielding a negative result for the TCRγ gene and a single band for the IgH gene indicating a monoclonal result (Fig. 5A, B, C). In both samples, Cµ with about 130 bp served as PCR positive control (Burnett et al., 2003). The IgH products centred around 120 bp (Burnett et al., 2003). As TCRγ positive control (Fig. 5D) we used the OSW T-cell line showing an oligoclonal band centred on 90 bp (Kisseberth et al., 2007).

### Establishment and Morphology of the CLBL-1M Cell Line

**Figure 6:**
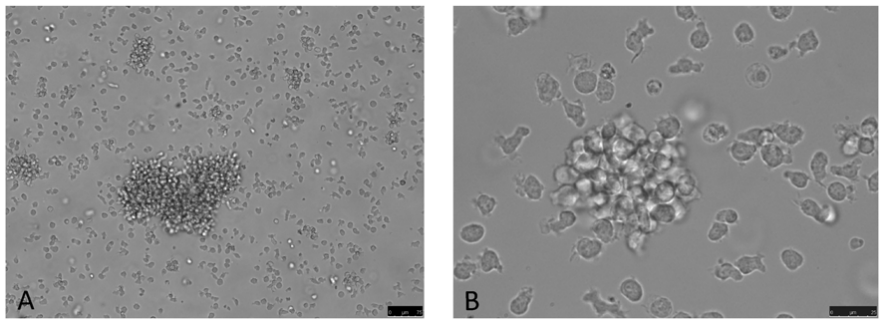
Morphologic appearance of non-adherent CLBL-1M cells. CLBL-1M cells form free floating clumps in the cell culture medium. Transmitted light images of 100x (A) and 400x (B) magnification.

**Figure 7:**
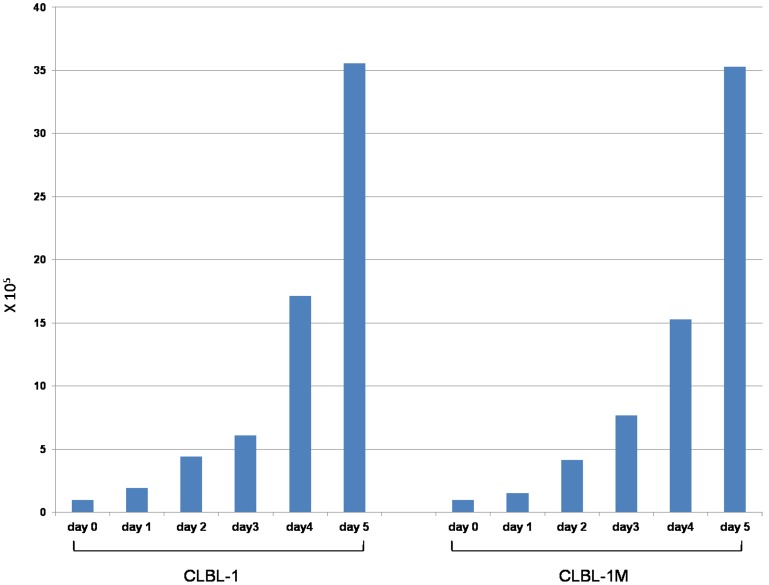
Proliferation doubling time of CLBL-1 and CLBL-1M on five consecutive days.

Initially, the cells derived from a mouse tumor showed an adherent appearance according to the initial growth status of the primary material and with the morphology and growth pattern resembling the original cell line CLBL-1. The primary prepared tumor cells were cultivated without the addition of supplementing growth factors. After 7 days of prolonged cultivation the cells started to proliferate in non-adherent floating clusters (Fig. 6). Up to now, the CLBL-1M cell line has been maintained in continuous culture for more than four months and showed a proliferation doubling time of 26.4 h during exponential growth under standard culture conditions (Fig.7).

### Immunophenotyping of the CLBL-1M Cell Line

FCM of the CLBL-1M cell line showed expression of MHCII+ (92.6%), and CD11a, and +CD79αcy+ (98.6%) (Fig. 4C). In addition cells were positive for CD45, CD45RA and negative for CD8, CD21, CD21-like antigen and CD56 (data not shown) Therefore they showed the same expression pattern as their mother cell line CLBL-1 published in Rütgen et al., 2010 [18]. The CLBL-1M cells were additionally negative for CD25 (Fig. 4C).

### Clonal IgH Gene Rearrangement of the CLBL-1M Cells

CLBL-1M were analysed by PCR for TCRγ and IgH gene rearrangements yielding a negative result for the TCRγ gene and a single band for the IgH gene indicating a monoclonal result (Fig. 5D). Cµ with about 130 bp served as PCR positive control (Burnett et al., 2003). The IgH products centred around 120 bp (Burnett et al., 2003). As TCRγ positive control (Fig. 5E) we used the OSW T-cell line showing an oligoclonal band centred on 90 bp (Kisseberth et al., 2007). These data confirm the stable IgH gene rearrangement shown in the initiating cell line CLBL-1.

### Cytogenetic Analyses of the Cell Line CLBL-1 and CLBL-1M

#### Cytogenetic investigation of CLBL-1

**Figure 8:**
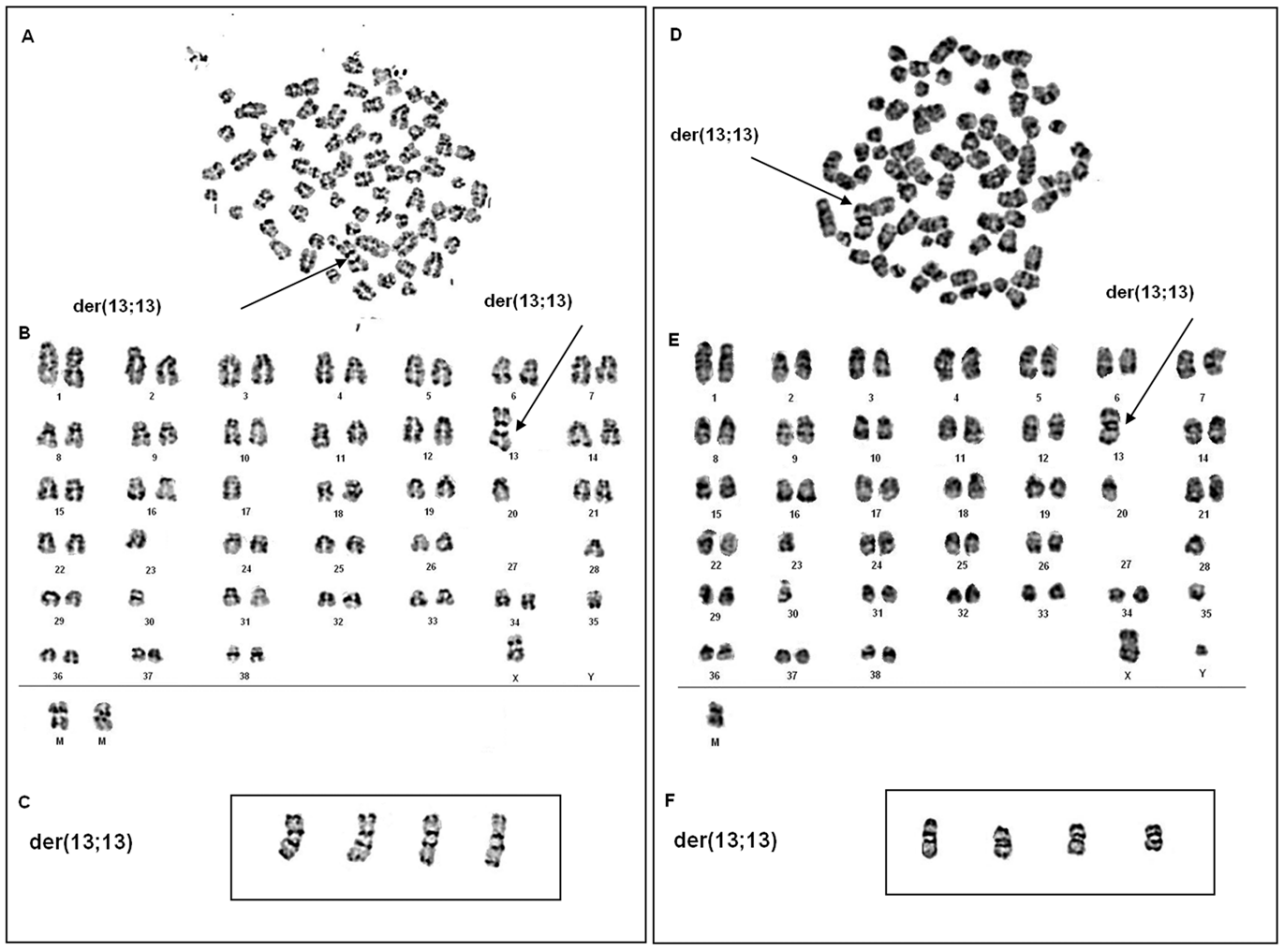
Metaphase spreads after GTG-banding from cells derived from CLBL-1 (A) and from CLBL-1M (D). Corresponding karyotypes of CLBL-1 (B) and CLBL-1M (E). The arrows indicate the derivative chromosomes der(13;13), comprising of two chromosomes 13 plus unidentified chromosomal material in the centromeric region. In the boxes four derivative chromosomes (der(13;13)) of CLBL-1 (C) and CLBL-1M (F) extracted from four different metaphases are shown.

Chromosome analyses of 21 metaphases revealed a canine hypodiploid karyotype with several chromosomal changes, including monosomies (e.g. monosomy 23, monosomy 30), derivative chromosomes (e.g. der(X)), and centric fusions (Fig. 8A+B). The chromosome number ranged between 69 and 73 (69 [1], 70 [8], 71 [9], 72 [2], 73 [1]), with 70 and 71 chromosomes being the predominant number. In 18 metaphases (85.7%) a bi-armed derivative chromosome (der(13;13)), consisting of two chromosomes 13 and unidentified chromosome material in the centromeric region, was observed. In Figure 8C four derivative chromosomes (der(13;13)) extracted from four different metaphases are shown.

### Cytogenetic Investigation of CLBL-1M

Cells for the cytogenetic investigation of CLBL-1M were derived from the injection-site located tumor tissue of a Rag2^−/−^γ_c_^−/−^ mouse. Chromosome analyses of 20 metaphases revealed a karyotype comparable to the CLBL-1 karyotype (Fig. 8D+E). For CLBL-1M, the chromosome number ranged between 70 and 73 (70 [1], 71 [17], 72 [1], 73 [1]), with clearly 71 chromosomes being the predominant number. The analyses revealed the same derivative chromosome (der(13;13)) as described for CLBL-1 in 19 metaphases (95%) (Fig. 8F).

### Quantitative Real-time PCR Analyses for *MYC* Expression in CLBL-1, CLBL-1M and DT08/40 Cells

**Figure 9:**
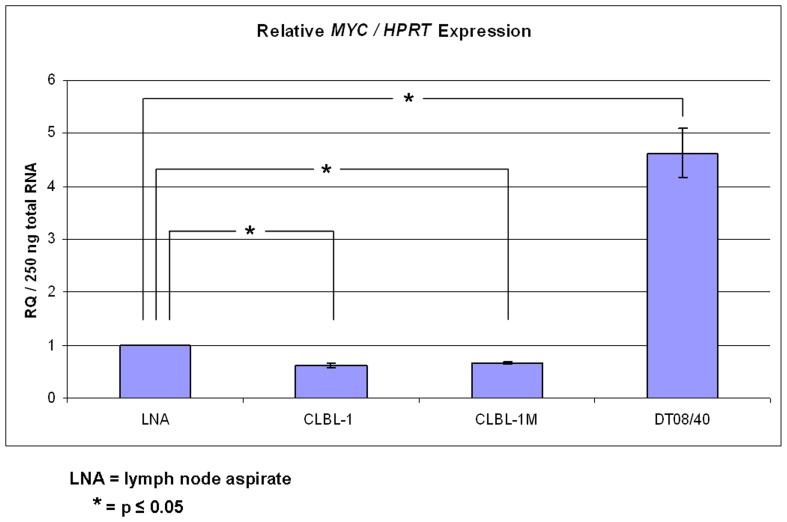
Relative *MYC/HPRT* expression in canine CLBL-1, CLBL-1M and DT08/40 cell lines and in a canine lymph node aspirate. Error bars are standard deviations. * indicates a statistical significant expression deregulation of the *MYC* gene when compared to non neoplastic control sample.

The non neoplastic lymph node sample was set as calibrator. *MYC/HPRT* expression levels for CLBL-1 and CLBL-1M varied from 0.621 (CLBL-1) to 0.662 (CLBL-1M) while the expression in DT08/40 showed 4.61 when compared to the calibrator (calibrator value was set as 1) (Fig. 9).

### Statistics of Relative Real-time PCR

*MYC* expression is down regulated using *HPRT* as endogenous control (housekeeping gene) in CLBL-1 (p = 0.000) and CLBL-1M (p = 0.000) when compared to the non neoplastic control sample. Relative *MYC* expression of DT08/40 in comparison to control sample is up regulated (p = 0.030).

### Proliferation Assay

Primary B-cells, B-cell lymphoid neoplasms and human chronic lymphatic leukemia (CLL) are reported to have a low *in vitro* mitotic activity, but the proliferation can be stimulated by incubation with the DSP30 CpG-oligonucleotide and IL-2 (Decker et al., 2000; Haferlach et al., 2007; Struski et al., 2009; Reimann-Berg et al., 2011). To examine if the initial canine B-cell line CLBL-1 as well as the Rag2^−/−^γ_c_^−/−^ mouse tumor-derived canine CLBL-1M cell line show as well a higher proliferation rate in response to DSP30 and/or IL-2 stimulation in different concentrations in comparison to the corresponding unstimulated cells the cell proliferative activity of the two cell lines was measured post stimulation with a standard BrdU proliferation test (Cell Proliferation ELISA BrdU (colorimetric), Roche). The incorporation of BrdU was assessed over a stimulation period of 24, 48, 72 and 96 h.

**Figure 10:**
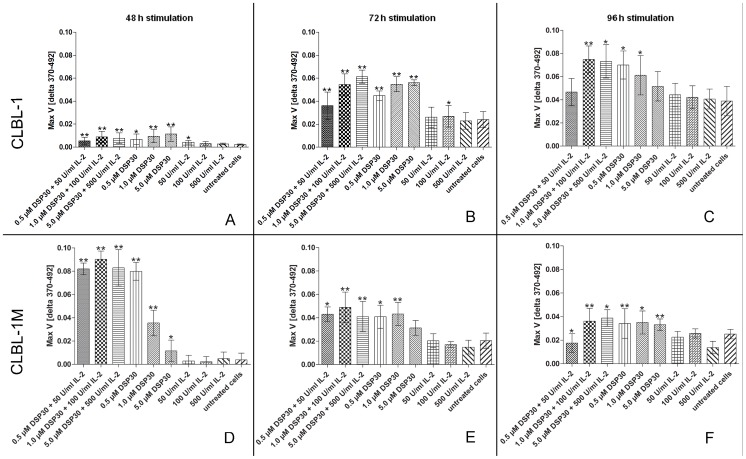
BrdU cell proliferation assay. Measured proliferation of CLBL-1 (A, B, C) and CLBL-1M (D, E, F) after stimulation with DSP30 and/or IL-2 at different time points (48 h, 72 h, 96 h) in comparison to untreated cells. Each bar represents a mean ± SD, *p≤0.05, **p≤0.001 to 0.01.

After a stimulation time of 24 h, no statistically significant data could be obtained in all different approaches stimulating CLBL-1 and CLBL-1M cells (data not shown). The BrdU incorporation assayed 48 h after stimulation of both cell lines was significantly increased in all samples stimulated with DSP30 and IL-2 in combination (Fig. 10A and 10D). The 48 h stimulation with DSP30 alone at all three concentrations (0.5, 1.0 and 5.0 µmol/l) led to a significant increase of CLBL-1 proliferation with highest effect at 5.0 µM (Fig. 10A) and for CLBL-1M cells at a concentration 0.5 µM DSP30 (Fig. 10D) while single IL-2 incubation for 48 hours only increased the proliferation significantly at a concentration of 50 U/ml in case of CLBL-1. After 72 h of stimulation with DSP30 and/or IL-2, a similar trend could be observed for the CLBL-1 as well as the CLBL-1M cell line (Fig. 10B and 10E). Merely the response of CLBL-1 cells to stimulation with 50 U/ml IL-2 is not significant anymore whereas a concentration of 100 U/ml IL-2 showed a significant effect after 72 h stimulation (Fig. 10B). Concerning CLBL-1M cells, the stimulation with 5.0 µM DSP30 has no longer a significant effect (Fig. 10E).

The measured proliferation of CLBL-1 cells after 96 h of stimulation revealed a decreased significance in all single experiments besides stimulation with 1.0 µM DSP30 and 100 U/ml IL-2 in combination, where a p<0.01 (**) is still given (Fig. 10C). Concerning CLBL-1M, the 96 h stimulation with combined DSP30+ IL-2 and with single applied DSP30 in all three concentrations resulted in significantly increased proliferative effects. In contrast, sole stimulation with IL-2 showed no significant effect on the cell proliferation.

## Discussion

The establishment of *in vivo* animal models and cell lines resembling human tumor diseases is a major tool in translational cancer research. Many cancers show strong similarities between the human and canine neoplastic disease including their histological appearance, tumour genetics, biologic behaviour and response to conventional therapies [26].

Lymphoma is one of these cancer types commonly affecting middle-aged individuals of both species, spontaneously. Due to the similarities in tumor behaviour and presentation the canine neoplasia is widely accepted to be a valuable model for human non-Hodgkin’s lymphoma [4], [26], [27].

In the present study, we describe the establishment and characterization of an *in vivo* lymphoma xenograft mouse model using a canine B-cell lymphoma cell line to analyse the tumorigenicity, genomic stability and histological and morphological similarity in comparison to the original material.

The used lymphoma cell line CLBL-1 [18] was derived from a canine diffuse large B-cell lymphoma (DLBCL) representing one of the most common lymphoma forms occurring in dogs and humans. The cells were inoculated subcutaneously to generate solid tumorous material at the injection site and for studying the homing of lymphoid-derived cells to the organs in the recipient mice. CLBL-1 *in vivo* application resulted in lymphoma-like disease and tumor formation.

All inoculated animals showed a diffuse foreign invasive population in solid tumor tissue, bone marrow, spleen, liver and uterus and ovaries detected by pathology, histopathology and immunohistochemical staining (IHC). Due to the fact that Rag2^−/−^γ_c_^−/−^ mice are alymphoid, we were not able to show neoplastic cells in lymph nodes or mediastinal mass. Rag2^−/−^γ_c_^−/−^ mice were selected due to their immunodeficient phenotype missing mature lymphocytes allowing the investigator to solely detect inoculated lymphocytes derived from the donor cell line.

Concerning immunohistochemical staining, CD79αcy was chosen as a responsible marker for B-lymphocytes focusing on the detection of the cells in liver, spleen, bone marrow, ovary and solid tumour tissue and since the chosen anti-human antibody crossreacts with canine cells [28]. In a former study characterising a canine lymphoma T-cell line (Kisseberth et al. 2007), *i.v.* injection of canine lymphoma cells in immunodeficient mice showed growth in the mesentery, pancreas, liver, spleen, lungs, peri-renal fat and small intestine. Our results using the herein described B-cell line parallel those findings. The infiltration and almost complete replacement of the normal cells with the malignant lymphocytic population in the reproductive organs was unexpected. To the best of our knowledge, this finding has not been previously reported in canine lymphoma. In human medicine, a quarter of the lymphomas arise in extranodal organs and about 1.5% of extranodal lymphomas primarily originate in the female genital tract [29], but also secondary involvement in case of high-stage lymphomas is possible and mostly affects the corpus besides cervix and ovaries [30]. The most commonly histological diagnosed type is the DLBCL [30]. These facts known in human medicine could probably explain the presence of the inoculated cells in the reproductive tract of the inoculated mice being characterized as a DLBCL and underline the aggressive infiltrating character of the established CLBL-1 cell line. Estrogene and progesterone receptors in lymphomas are known in humans and horses. In horses the presence of monoclonal antibodies to nuclear progesterone has been confirmed immunohistochemically (Henson et al. 2000). In canine lymphoma, these facts are discussed controversially (Teske et al. 1987; Vicini et al. 1991), but could be a reason for the presence of infiltration in the organs of the reproductive tract in the inoculated mice.

Flow cytometric cell surface and intracellular marker analyses of cells isolated from the tumor mass and the mouse tumor derived cell line CLBL-1M revealed the same immunophenotypical characteristics represented by the same antigen expression in comparison to the original CLBL-1 cell line. The cell surface and intracellular markers have been chosen according to the original marker panel used to characterise the parental CLBL-1 cell line. The cells derived from the tumor mass and the CLBL-1M cell line stained positive for CD11a, CD79αcy, MHCII and negative for CD3 and CD5. The CLBL-1 and the CLBL-1M cell line show nearly identical *in vitro* growth characteristics including proliferation doubling times represented by 26.4 h in the CLBL-1M cells. This result is comparable to the published doubling time of the CLBL-1 cells showing 31 h [18]. These gained results confirm the phenotype of the parental CLBL-1 cell line leading to the conclusion that the growth status and the antigen expression remained stable during tumor formation in the xenograft model.

On the genomic level, the polymerase chain reaction for B- and T-cell antigen receptor rearrangement (PARR) analysis of the xenograft material revealed a clonal band for the IgH gene rearrangement in all samples thus confirming the primordial origin of the tumorous material and further providing evidence for its stable phenotype.

The importance of non-random cytogenetic abnormalities in human leukaemia and lymphoma has been recognised since the early eighties [31]. Several reports about cytogenetic analyses of canine lymphoid neoplasms showed that lymphomas in dogs are characterised by non-random cytogenetic abnormalities as well [8], [12], [16], [32].

To examine potential cytogenetic changes during tumor induction in Rag2^−/−^γ_c_^−/−^ mice, the initial CLBL-1 B-cell lymphoma cell line as well as the CLBL-1M cell line established from a tumor located at the injection site of a Rag2^−/−^γ_c_^−/−^ mouse were cytogenetically analysed and compared. We observed relatively stable modal chromosome copy numbers in CLBL-1M compared to CLBL-1. Both expressed the same prominent derivative chromosome (der(13;13)) and CLBL-1M did not acquire further chromosomal alterations. Thus we conclude that chromosomal stability was maintained. A polysomy of CFA 13 could not be detected but aberrations of CFA 13 were described to be involved in several canine neoplasias including lymphomas [9], [12], [16]. Interestingly, the canine orthologue to *c-MYC* gene is coded on CFA13 and thus aberrations affecting this chromosome could be affecting the expression of the gene. Comparative real-time PCR analyses of *MYC* expression in CLBL-1, CLBL-1M, a non neoplastic lymph node sample, and DT08/40 (a canine prostate cancer cell line showing polysomy of CFA13) showed that -in contrast to DT08/40- both cell lines do not over-express *MYC*. CLBL-1 and CLBL-1M showed a statistical relevant down-regulation of *MYC* expression. However, this difference in expression is in a minor range, probably explainable by regular biologic variance. In contrast to this, the clear over-expression of *MYC* in DT08/40 indicates that the several copies of CFA13 in this cell line correlate with *MYC* over-expression. As we were not able to identify polysomies of CFA13 in CLBL-1 and CLBL-1M the obtained qPCR data matches the karyotype analysis.

In general, conventional cytogenetic analyses of B-cell lymphoid proliferations are difficult to perform due to a low mitotic activity of the B-cells. Therefore, mitogen stimulation of B-cells is required to gain a sufficient number of metaphases for analyses. For human chronic lymphatic leukemia (CLL) and B-cell lymphoid neoplasms, the immunostimulatory CpG-oligonucleotide DSP30 in combination with interleukin-2 (IL-2) has been reported to be an easy and efficient stimulus for metaphase generation [33], [34], [35]. As recently reported, B-cells from a canine high-grade lymphoma could successfully be stimulated with concentrations of 1.0 µM DSP30 and 100 U/ml IL-2 for 72 h resulting in an adequate number of metaphases [15].

To examine if the initial B-cell line CLBL-1 as well as the CLBL-1M cell line derived from a Rag2^−/−^γ_c_^−/−^ mouse tumor respond in the same dose-dependency as primary B-cells to DSP30 and IL-2 stimulation with a higher mitotic rate, the two cell lines were incubated with DSP30 and IL-2 to measure the proliferation subsequently by BrdU incorporation. The highest significant stimulatory effect on CLBL-1 cells was reached with the combined application of DSP30 and IL-2 after 72 h stimulation. Concerning CLBL-1M stimulation, the highest effects were also measured with DSP30 and IL-2 in combination, but at a stimulation time of 48 h. After 72 h of stimulation, CLBL-1M showed similar proliferative responses as CLBL-1 to DSP 30 and/or IL-2 stimulation. Merely the combination of 5.0 µM DSP30+500 U/ml IL-2 and the single stimulation with 5.0 µM DSP30 seems less effective on CLBL-1M cells. Considered in total, the five-fold higher combination of the stimulating agents (5.0 µM DSP30+500 U/ml IL-2) showed partially a slightly higher effect, but the difference to the stimulation with 1.0 µM DSP30+100 U/ml IL-2 is marginal and the cost-benefit ratio needs to be evaluated case-dependently. Differences in cell response to the stimulating agents could be explained by a differential expression of the relevant receptors as e.g. CD25. FCM analyses for CD25 expression in the analysed cell lines revealed negative results indicating that the observed different stimulation response at 48 h is probably mediated by an alternative way. However, in conclusion we could demonstrate that the immunostimulatory CpG-oligonucleotide DSP30 in combination with IL-2 results in higher proliferation rates of CLBL-1 and CLBL-1M cells and confirm the findings of Reimann-Berg et al. in primary material [15] with respect to the applied concentrations of 1.0 µM DSP30 and 100 U/ml IL-2 in combination.

The results show that during the tumor formation in the Rag2^−/−^γ_c_^−/−^ mouse model and subsequent *in vitro* cell culture, the CLBL-1 cell line did not lose the capability to respond in the same way as freshly taken, short term cultured B-cell lymphoma cells.

In the present study we could show that the canine cell line CLBL-1 is capable of inducing tumor (lymphoma) formation in mice. These tumors are morphologically and phenotypically similar to the canine end-stage disease. This fact makes the model an interesting candidate for further investigations into the diagnosis and therapy of canine lymphoma and has the potential to promote the translational and comparative lymphoma research in humans and dogs.
